# Role of Nitrogen and Yttrium Contents in Manufacturing (Cr, Y)N_x_ Film Nanostructures

**DOI:** 10.3390/nano12142410

**Published:** 2022-07-14

**Authors:** Raira Chefer Apolinario, Alisson Mendes Rodrigues, Pedro Renato Tavares Avila, Júlia Nascimento Pereira, Carlos Alberto Ospina Ramirez, Philipp Daum, Fabiana Pereira da Costa, Hélio de Lucena Lira, Gelmires de Araújo Neves, Christian Greiner, Haroldo Cavalcanti Pinto

**Affiliations:** 1 São Carlos School of Engineering—EESC, University of São Paulo—USP, São Carlos 13563-120, SP, Brazil; raira.apolinario@usp.br (R.C.A.); pedrorenatoavila@gmail.com (P.R.T.A.); julianp@usp.br (J.N.P.); 2 Laboratory of Materials Technology (LTM), Department of Materials Engineering, Federal University of Campina Grande (UFCG), Campina Grande 58429-900, PB, Brazil; alisson.mendes@professor.ufcg.edu.br (A.M.R.); fabiana.costa@estudante.ufcg.edu.br (F.P.d.C.); helio1309@gmail.com (H.d.L.L.); gelmires.neves@ufcg.edu.br (G.d.A.N.); 3 Nanomaterials Division, Brazilian Nanotechnology National Laboratory—LNNano Brazilian Center for Research in Energy and Materials—CNPEM, Campinas 13083-100, SP, Brazil; carlos.ospina@lnnano.cnpem.br; 4 IAM-ZM MicroTribology Center µTC, Strasse am Forum 5, 76131 Karlsruhe, Germany; philipp.daum@iwm.fraunhofer.de (P.D.); christian.greiner@kit.edu (C.G.); 5 Karlsruhe Institute of Technology (KIT), Institute for Applied Materials (IAM), Kaiserstrasse 12, 76131 Karlsruhe, Germany

**Keywords:** (Cr, Y)N_x_, HiPIMS, nitrogen flow rate, wear protection

## Abstract

The high-power impulse magnetron sputtering (HiPIMS) technique was applied to deposit multilayer-like (Cr, Y)N_x_ coatings on AISI 304L stainless steel, using pendular substrate oscillation and a Cr-Y target and varying the nitrogen flow rate from 10 to 50 sccm. The microstructure, mechanical and tribological properties were investigated by scanning and transmission electron microscopy, X-ray photoelectron spectroscopy, X-ray diffraction, instrumented nano-hardness, and wear tests. The columnar grain structure became highly segmented and nanosized due to pendular substrate oscillation and the addition of yttrium. The deposition rate increased continuously with the growing nitrogen flow rate. The increase in nitrogen flow from 10 to 50 sccm increased the hardness of the coatings (Cr, Y)N_x_, with a maximum hardness value of 32.7 GPa for the coating (Cr, Y)N_x_ with a nitrogen flow of 50 sccm, which greatly surpasses the hardness of CrN films with multilayer-like (Cr, Y)N_x_ coatings architecture. The best mechanical and tribological performance was achieved for a nitrogen flow rate of 50 sccm. This was enabled by more elevated compressive stresses and impact energies of the impinging ions during film growth, owing to an increase of HiPIMS peak voltage with a rising N2/Ar ratio.

## 1. Introduction

Nowadays, advances in sustainable energy and technologies are critically dependent on designing materials with improved properties. In another way, thin-film development is essential for discovering and improving new technologies [1]. Studies on the growth mechanisms of thin films deposited using sputtering techniques have led to innovation and a better comprehension of the fundamental aspects and the physicochemical and technological properties of coatings, in addition to improvements in the process technologies [1,2,3].

The physical vapor deposition technique is widely explored in various industrial areas and used in several coating depositions. Magnetron sputtering is a plasma-based coating technology in which inert gas atoms (usually Ar) are ionized and accelerated due to the potential difference between the negatively polarized target (cathode) and the anode. Hence, the interaction of ions with the surface of the target causes the ejection of ionized atoms that travel and condense on the substrate, forming the thin film [2,3,4].

High-power magnetron sputtering (HiPIMS) is based on creating a high-density plasma using a sputtering source, increasing peak power with short pulses [5,6]. The high electron density generated by this type of discharge increases the ionization probability by colliding electrons from atoms that have been sprayed, contributing to the increase of the ion-to-neutral-species ratio in the plasma. [7,8]. The HiPIMS tech Due to its high ionization rate, the HiPIMS technique improves the integrity of hard coatings, including the density and adhesion to the substrate growth and condensation of thin films, which usually occurs in conditions far from thermodynamic equilibrium [9,10,11,12]. However, controlling the growth of thin films during the deposition process requires heating the substrate. The temperature gradient during the deposition process changes the energy transferred to the coating-forming elements (adatoms); this energy is decisive for the activation of surface and bulk diffusion processes that allow for control over the film’s morphology [12].

Yttrium (Y) is a rare earth element that has been widely used to improve the strength of materials. Yttrium is known to segregate to grain boundaries, thus pinning them, limiting the main oxygen diffusion pathways, and increasing the oxidation resistance of the material [13]. For these reasons, adding Y is desirable for protective coatings used in high-temperature applications, such as thermal barriers, cutting tools, and piston rings. A small percentage of yttrium (<2 atom %) allows coatings of transition metal nitrides to remain in the fcc-NaCl structure, changes the fiber texture to [100]//ND, and is the connection (“plugging”) of grain boundaries that inhibits the diffusion of cations out of the coating towards the free surface by changing the driving force of oxidation [13,14]. These effects are beneficial for high-temperature applications and do not compromise the mechanical properties [15].

The properties of coatings can be modified by changing HiPIMS process parameters such as bias polarization, working pressure, gas ratio, substrate displacement, and impinging angle of ion bombardment [9,16,17,18,19,20,21]. The microstructure, mechanical and tribological behavior are strongly related to N_2_/Ar flow ratios during deposition. Different nitrogen contents cause changes in the crystal lattices and width of the constituent columns growing perpendicular to the substrate [22,23]. CrN coatings exhibit interesting electrical and magnetic properties due to semiconductor behavior and magnetic ordering, thus becoming a promising coating in magnetic semiconductor thinner applications [6,22,24,25,26].

This work is based on the influence of nitrogen and yttrium additions on the microstructure, mechanical and tribological properties in the microstructural, mechanical, and tribological analysis of multilayer (Cr, Y)Nx coatings deposited by HiPIMS on AISI 304L stainless-steel substrates, using Dynamic Glancing Angle Deposition (DGLAD); DGLAD is a manufacturing route to develop nanostructured coatings with increased wear resistance and high hardness and toughness values due to systematic nanostructured layer formation [9,18,19,20,21].

## 2. Materials and Methods

### 2.1. Coating Deposition Process

The (Cr, Y)N_x_ coatings manufactured in this work were deposited on AISI 304L stainless-steel substrate disks (30 mm diameter × 10 mm thick). Previously, the 304L stainless-steel substrates were sanded with SiC sandpaper; polished with diamond suspensions and colloidal silica; cleaned in ultrasonic baths, with acetone, before the deposition; and blow-dried.

**Figure 1:**
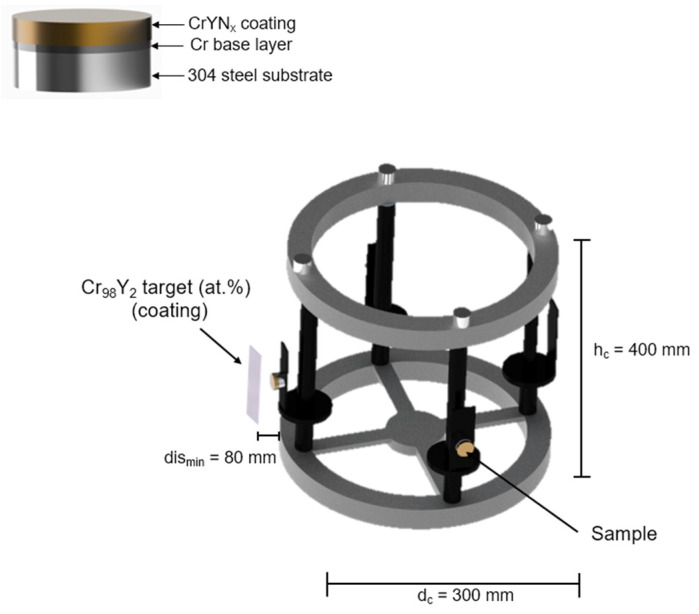
Schematic representation of the sputtering system using CrY target (98–2 at.%) and 304L substrates for deposition of (Cr, Y)N_x_ coatings varying the nitrogen flow. The minimum distance (dis_min_) between target and substrate surface is 80 mm, carousel height (h_c_) is 400 mm, and carousel diameter (d_c_) is 300 mm.

A CrY alloy target (98–2 at.%, 220 mm × 110 mm × 12 mm) was used for film deposition with a distance between the substrate and target of 80 mm in the 0° position. When oscillating to 15°, the distance between target and substrate is 119 mm, as shown in Figure 1, and connected to the power supply of HIPIMS. The deposition experiments were performed using a sputtering spray system consisting of a HiPIMS TruPlasma 4004 (TRUMPF Hüttinger, Ditzingen, Germany) feed source installed in a reaction chamber—HiPIMS-250 (Plasma LIITS, Sao Paolo, Brazil) of octagonal shape with inner chamber dimensions of 600 mm base diameter and 660 mm height. A Pinnacle MDX power supply (Advanced Energy, Fort Collins, CO, USA) was added to supply −180 V substrate bias voltage. The sample holder performs oscillatory movements in front of the target, with a time interval of 30 s and an angular period of ±15°. The substrates were allowed to oscillate with an amplitude of ±15°, with position 0° corresponding to the sample surface parallel to the target surface. The entire cycle from 0 to −15° to +15° and back to 0° took 120 s, with a nominal angular speed of approximately 0.6 rpm. The (Cr, Y)N_x_ coatings depositions were carried out in an Ar atmosphere, at a constant flow rate of 50 sccm and varying nitrogen flow (10, 20, 30, 40, and 50 sccm), except for ion etching and base layer, where it was under an atmosphere of Ar only with 50 sccm. The working pressure was set to 0.266 Pa at 400 °C, and a substrate bias of −180 V was applied. The HiPIMS pulse frequency was 500 Hz, and the ton was 200 µs. Initially, an ion etching with Cr^+^ ions was carried out for 1 h, using HiPIMS with an average peak power density of 3.0 W/cm^2^, 100 Hz, and 50 µs of ton and substrate polarization of −800 V, thus allowing for cleaning and superficial ionic implementation. Subsequently, the deposition of the (Cr, Y) base layer was carried out for twelve minutes, using the same substrate bias of −180 V applied later during the deposition of the (Cr, Y)N_x_ films. The parameters for deposition of (Cr, Y)N_x_ were kept constant: the peak power HiPIMS density was 90 W/cm^2^, the peak current density over the target area was 203 mA/cm^2^, and deposition was 2 h. Such experimental conditions were employed in agreement with our previous work [20].

### 2.2. Coating Characterization

The morphology of the multilayer-like (Cr, Y)N_x_ coatings was investigated using a Field Emission Scanning Electron Microscope FEG-Inspect, F-50 (FEI, Eindhoven, The Netherlands). Scanning Ion Microscopy (SIM) was also performed at 30 kV and 18 pA (Thermo Fisher Scientific, Waltham, MA, USA), using an FEI—Helios 650 dual-beam equipment. Electron microscopy characterization techniques (Transmission Electron Microscopy—TEM, Scanning Transmission Electron Microscopy—STEM, Selected Area Electron Diffraction—SAED, Energy-Dispersive X-ray Spectroscopy—EDS) were carried out on a JEM-2100F (JEOL Ltd., Tokyo, Japan) and a Double Spherical Aberration Corrected Titan Cubed Themis (Thermo Fisher Scientific) microscope, at 200 and 300 kV, respectively, at the LNNano. Cross-section lamellae were prepared by Focused Ion Beam (FIB) on a Dual Beam Scios 2 (Thermo Fisher Scientifics) microscope at the LNNano. Roughness measurements and surface mapping were performed in tapping mode using NanosurfFlex Atomic Force Microscope (AFM) (Nanosurf, Liestal, Switzerland).

X-ray photoelectron spectroscopy (XPS) was performed to determine chemical depth profiles using a Versaprobe PHI 5000 (Chanhassen, MN, USA), using 0.2 eV energy resolution and 15 eV Al kα radiation and an area with a diameter of approximately 200 µm.

X-ray diffraction (XRD) was applied to identify phases and residual stress in (Cr, Y)N_x_ films. All XRD patterns were conducted on a Panalytical MRD-XL X-ray diffractometer (Almelo, The Netherlands), using Mo Kα radiation (0.7093 Å).

Nanohardness measurements were performed with a PB1000 mechanical tester (Nanovea, Irvine, CA, USA). A load of 50 mN was used. The nanohardness measurements were performed with a Berkovich dyad tip for each (Cr, Y)N_x_ coating. Using the Oliver–Pharr [27] relationship, the average hardness and elastic modulus, along with standard deviations, were calculated.

Wear tests were performed by linear reciprocal sliding in accordance with ASTM G113, using the PB1000 mechanical tester. For the wear test, Al_2_O_3_ spheres were used as a counter body; the tests were performed without lubrication and at room temperature. The normal load was 5 N, and the sliding speed was 60 mm/s along a 1 mm–long track. Five hundred cycles were performed, and, at the end of the measurement, the slip distance was 1000 mm. Subsequently, the wear measurements in the (Cr, Y)N_x_ films were measured by using a non-contact optical profilometer, in which six cross-section profiles were obtained according to ASTM G133-05, and we obtained the average area of material loss from the area of the calculated cross-section, which, when multiplying by the sliding distance, provided the wear volume; the surfaces of the Al_2_O_3_ counter body were also measured, and for the analysis, the theoretical sphere was subtracted to observe the material adhered to the sphere’s surface.

## 3. Results and Discussion

**Figure 2:**
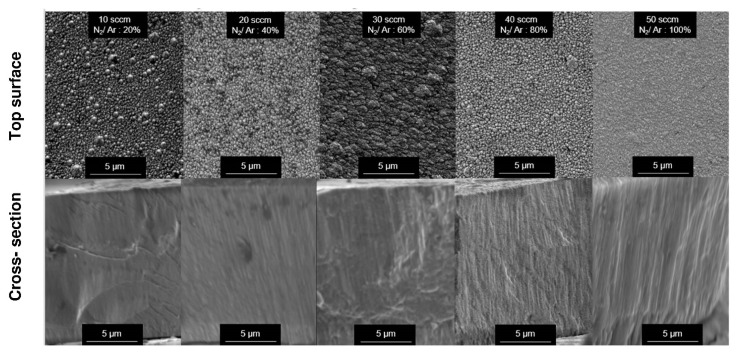
FEG–SEM micrographs of the top surface and fractured cross-sections were observed in (Cr, Y)N_x_ coatings deposited by HiPIMS as a function of the N_2_ flow rate.

**Figure 3:**
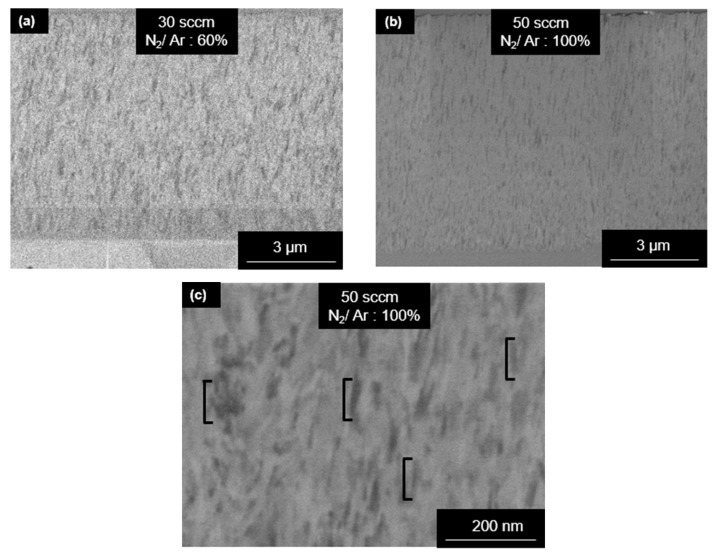
SIM micrographs of (Cr, Y)N_x_ coatings’ cross-sections with a nitrogen flow rate of (**a**) 30 sccm and (**b**) 50 sccm. (**c**) Grains are 100 to 500 nm long for a nitrogen flow rate of 50 sccm.

**Figure 4:**
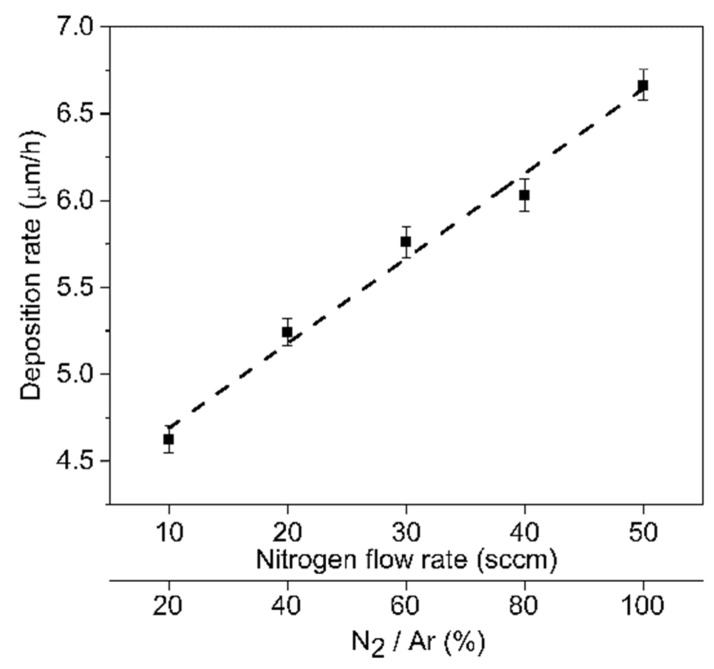
Deposition rate as a function of N_2_ flow rate.

FEG–SEM micrographs were obtained of the top surface and cross-section of the (Cr, Y)N_x_ coatings fabricated with different N_2_/Ar flow rates. From the top surface micrographs (Figure 2), one notices that, by increasing the nitrogen flow rate, a slight reduction in the size of the columnar structure is observed. The (Cr, Y)N_x_ films in the fracture cross-section showed a dense columnar microstructure (Figure 2), which becomes segmented due to the oscillatory motion of substrates. The grains with the columnar structure are 100 to 500 nm for a nitrogen flow rate of 50 sccm. As the nitrogen flux increased, an increase in thickness was observed, having the m thickness at 50 sccm, as demonstrated by scanning ion microscopy (SIM) (Figure 3). Nitrogen flow was responsible for grain growth; as the nitrogen flow rate increased, the thickness increased and reached its maximum value with a flow of 50 sccm (see Figure 2 and Figure 4). Such behavior is because the negative HiPIMS peak voltage increases (from −718 to −757 V) with the N_2_ flow rate, and, consequently, the ion bombardment is enhanced. The deposition rate and stress increase show that the coating deposition (Cr, Y)Nx is in metallic mode [28]. The base layer was the average oscillation of the nanostructure, which was around 230 nm, which is expected when using ±15° and an oscillation period of 120 s.

**Figure 5:**
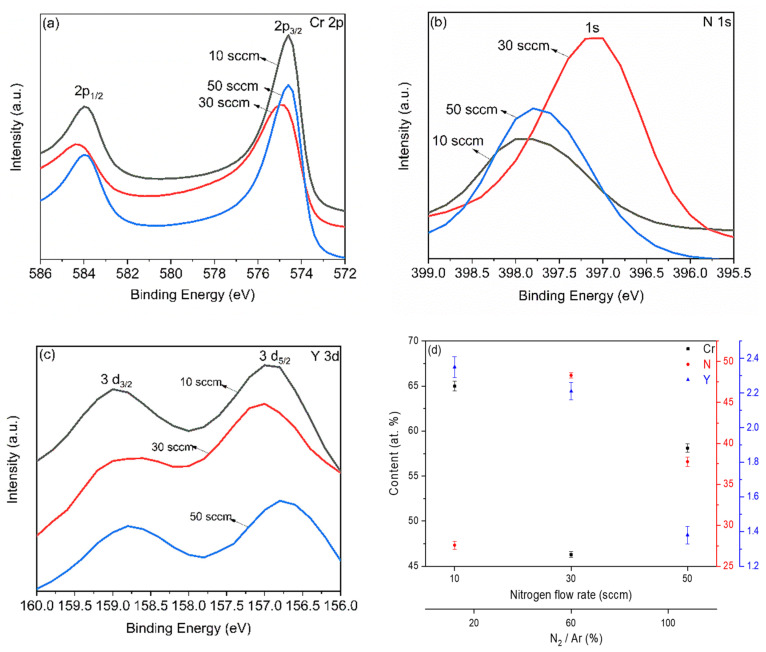
XPS spectra of Cr2p (**a**), N1s (**b**), and Y3d (**c**), and spectra of atomic concentrations as a function of nitrogen flow rate (**d**).

The XPS technique was used to study the chemical composition of the multilayer-like (Cr, Y)N_x_ films. The XPS spectra of Cr2p and N1s binding energies are displayed in Figure 5a–c for the (Cr, Y)N_x_ films fabricated under different N_2_ flow rates (10, 30, and 50 sccm). The Cr2p spectra contain two peaks, 2p_1/2_ and 2p_3/2_ (Figure 5a). There was no significant difference in the binding energy of the 2p_1/2_ peak (theoretical peak at 584 eV), verified at 583.9 eV for the N_2_ flow rates of 10 and 50 sccm and 584.2 eV for 30 sccm. Similarly, the 2p_3/2_ peak (theoretical peak at 575 eV) was observed at approximately 574.6 eV for all the nitrogen flow rates studied [6,29,30]. In contrast, the Cr2p peak intensities, which correspond to the Cr content in the films, are at the maximum for 10 sccm, decrease to a minimum at 30 sccm, and increase again for 50 sccm. At an N_2_ flow rate of 10 sccm, the Cr2p binding energies are higher due to the formation of both (Figure 5a) CrN (theoretical peaks at 575 eV) and Cr_2_N (theoretical peaks at 576.4 eV). These higher values are due to the greater distance between the Cr, Y, and N atoms, decreasing the bond strength between the elements. The nitrogen atoms can be placed in positions previously occupied by the Cr atoms.

Figure 5b shows the spectra of N1s, and there is a single broad peak with significant variations in peak intensity. The N_2_ flow rate of 30 sccm leads to maximum nitrogen content, while 10 sccm promotes a minimum and 50 sccm, an intermediate nitrogen concentration. The N1s peaks lie at about 397.4 eV for all nitrogen flow rates, i.e., 10, 30, and 50 sccm. This binding energy is associated with the transition metal nitride CrN, exhibiting the theoretical peak at 396.7 eV. The lower binding energies for 10 and 50 sccm can be attributed to the increased distance between the Cr, Y, and N atoms and the decreasing atomic bonding. The N atoms can be placed in positions previously occupied by the Cr atoms [6,29]. For the Y3d spectra (Figure 5c), two peaks, 3d_3/2_ and 3d_5/2_, slightly shifted. The 3d_3/2_ and 3d_5/2_ binding energies lie at approximately 158.8 and 156.6 eV, respectively, compared to the theoretical values of 160 and 158 eV. This small energy shift is due to the (Cr, Y)N_x_ coatings bond [30,31].

From the spectra of atomic concentrations of Cr, Y, and N as a function of nitrogen flux (see Figure 5d), we analyzed that nitrogen concentrations increase in (Cr, Y)N_x_ films from 10 to 50 sccm. However, the nitrogen flow rate of 30 sccm leads to a maximum atomic concentration of nitrogen. This can be attributed to increasing the nitrogen flow. For the (Cr, Y)N_x_ coatings deposited by HiPIMS, the flux of the ionized species increases as the negative peak voltage of the HiPIMS increases (from −718 to −757 V) [30].

**Figure 6:**
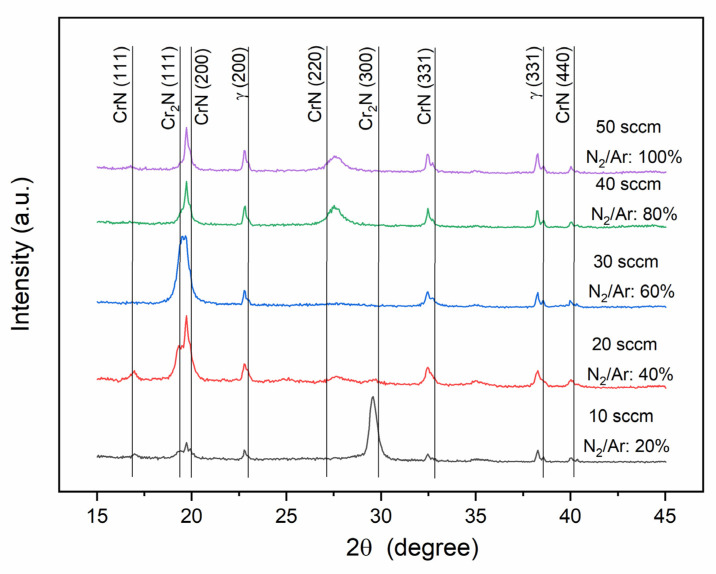
θ-2θ X-ray diffractograms of the (Cr, Y)N_x_ coatings function of nitrogen flow rate.

Figure 6 displays X-ray diffractograms of (Cr, Y)N_x_ films deposited by HiPIMS under different N_2_ flow rates (10, 20, 30, 40, and 50 sccm). For the film grown with an N_2_ flow rate of 10 sccm, one can mainly note the presence of the hexagonal phase Cr_2_N with a (100) preferential orientation in the normal surface direction (ND). This agrees with the XPS analyses that verified higher binding energies for the Cr2p photoelectrons as a result of Cr_2_N occurrence for the 10 sccm nitrogen flow rate. With a nitrogen flow rate of 20 sccm, the (300) peak of Cr_2_N remains broad but becomes small, thus indicating a low volume fraction of the nanostructured hexagonal phase. According to previous works [27,29], it was analyzed that the nitride peaks tend to widen as the flow of N2 gas is reduced. A face-centered cubic phase (fcc-CrN) also occurs with five diffraction lines, i.e., (111), (200), (220), (311), and (440). Fcc-CrN is encountered for the different nitrogen flow rates and is the major phase for all flow rates above 10 sccm. The preferred growth orientation is (100) for all N_2_ flow rates. This is desired for (Cr, Y)N_x_ coatings under situations of high atomic mobility and a low percentage of yttrium (<2 at.%) [27,29]. The increase in nitrogen flow rate of the N_2_ ratio according to Reference [29] was also analyzed for favoring preferential growth orientation in (100) caused by an increase in ion density. In addition, the diffractograms revealed peaks from the austenitic stainless-steel substrate (Figure 6), and Table 1 displays the XRD data of the (Cr, Y)N_x_ films, such as domain size, planes, and orientation. Table 1 agree with Figure 2 and Figure 3 that the microstructures formed with a columnar grain structure have domain sizes between approximately 50 and 500 nm, and the domain size increases with the increasing nitrogen flow rate in (CrY)Nx coatings.

**Figure 7:**
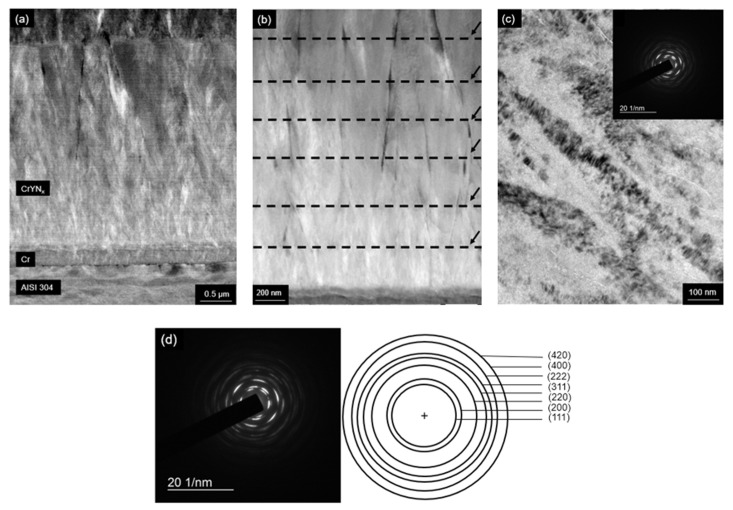
HAADF-STEM (**a**) and BF TEM (**b**) Nanostructured multilayer-like (Cr, Y)N_x_ coating with nitrogen flux of 50 sccm (**c**) images of the coating deposited by HiPIMS, with an N_2_ flow rate of 50 sccm (**d**) SAED image and scheme of its identification of a multilayer-like (Cr, Y)N_x_ coating.

**Figure 8:**
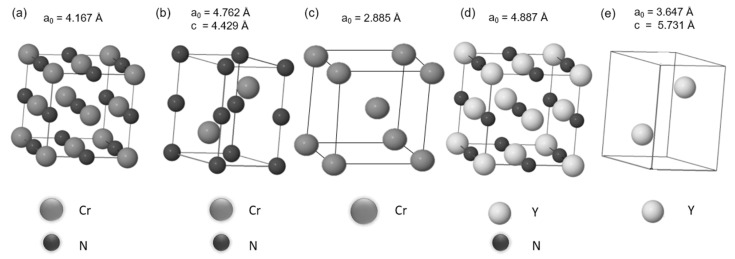
Schematic illustrations of crystal structures for (**a**) fcc cubic CrN, (**b**) hexagonal Cr_2_N, (**c**) bcc cubic Cr, (**d**) fcc cubic YN, and (**e**) hexagonal Y.

The High-Angle Annular Dark-Field Scanning Transmission Electron Microscopy (HAADF-STEM) image of the cross-section shows the morphology of the Cr base layer and the (Cr, Y)N_x_ films with a highly segmented columnar structure (Figure 7a). Figure 7b shows (Cr, Y)Nx multilayer nanostructure with N_2_ flux of 50 sccm. So that Figure 7b shows that columnar grains exhibit a zig-zag grain growth morphology (black arrows) that is consistent across multiple layers. Bright-Field Transmission Electron Microscopy (BF-TEM) image of the nanostructured film grown with a nitrogen flow rate of 50 sccm and oscillatory motion of the substrate also shows the columnar feature of the deposition (Figure 7c). The Selected Area Electron Diffraction (SAED) patterns of the (Cr, Y)N_x_ films confirm the formation of nanosized grains that generate the rings in the Diffraction Patterns (DPs), as shown in Figure 7d. Figure 8b–e shows schematic illustrations of the crystal structures of the Cr_2_N, CrN, Cr, YN, and Y phases. The CrN based coatings are characterized by their fine granulation and dense structure, and the thickness of the coatings is related to peak voltage, which corroborates the (Cr,Y)Nx coatings in that increasing the nitrogen flux increases the thickness of the coating by increasing the peak voltage [20].

**Figure 9:**
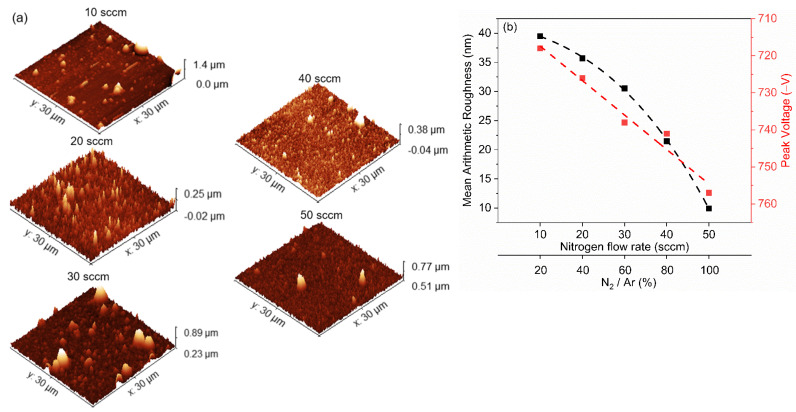
(**a**) Surface morphology maps obtained by tapping-AFM of (Cr, Y)N_x_ films deposited by HiPIMS as a function of N_2_ flow rate. (**b**) Evolution of mean surface roughness and corresponding effects on peak voltage as a function of N_2_ flux.

The surface maps obtained by tapping-AFM of the (Cr, Y)N_x_ films deposited by HiPIMS under substrate oscillation are presented in Figure 9a. The coating surface for all N_2_ flow rates exhibited cone-shaped growth-defect particles that showed the smallest amount for the 50 sccm conditions. Generally, these growth defects come from impurity particles still located on the substrate or the surface of the growing multilayer-like (Cr, Y)N_x_ coatings during the onset of deposition. The impact of the N_2_ flow rate on the arithmetic mean of surface roughness (Ra) and the corresponding effect on peak voltage HiPIMS is seen in Figure 9b. As the nitrogen flow rate increases from 10 to 50 sccm, the surface roughness decreases, while the negative peak voltage increases from 718 to 757 V during HiPIMS pulses.

**Figure 10:**
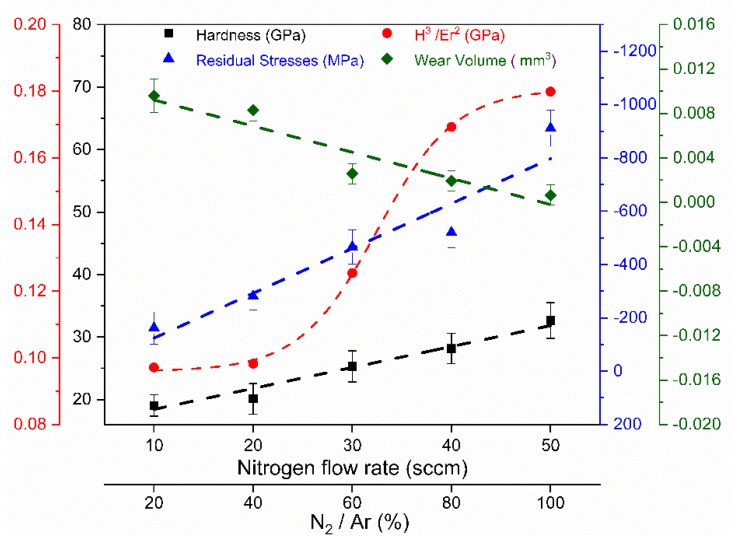
Mechanical properties and wear for multilayer-like (Cr, Y)N_x_ coatings.

Figure 10 shows the mechanical and tribological properties from which it is possible to observe an evolution of the residual stresses in the multilayer-like (Cr, Y)N_x_ coatings deposited by HiPIMS as a function of N_2_ flux. All coatings exhibited compressive residual stresses. By increasing the N_2_ flow rate from 10 to 50 sccm, residual stresses increased (from −162 to −911 MPa). Residual stresses in magnetron-sprayed coatings often arise due to intrinsic growth and thermal stresses [27]. The compressive stresses gradually grew with increasing nitrogen content because of rising negative peak voltages. Hence, incorporating nitrogen in the coatings at a higher flow rate can effectively reduce surface roughness and cone-shaped defects and increase the compressive stresses.

When the N_2_ flow rate increases, the hardness values also increase, see Figure 10. The highest hardness value (32.6 ± 2.9 GPa) was measured to the N_2_ flow rate equal to 50 sccm. This is caused by the highest peak voltage during sputtering and the maximum compressive stresses, despite a nitrogen content lower than 30 sccm. There was a positive correlation between the rising N_2_ flow ratio (from 10 to 40 sccm) and the hardness values, which increased from 19.0 ± 1.7 to 28.1 ± 2.4 GPa, and the elastic modulus, which increased from 266.4 ± 59.0 to 362.8 ± 54.3 GPa due to the replacement of Me-Me bonds by Me-N bonds in the (Cr, Y)N_x_ film [32,33,34,35]. The hardness values obtained are higher than those of pure multilayer-like CrN coating in Reference [20] that was grown with 500 Hz and exhibited a maximum hardness of 26 GPa. This increase was due to the positive influence of yttrium on the microstructure of the fcc-CrN phase, which was considerably refined.

To evaluate the suitability of (Cr, Y)N_x_ coatings for tribological applications, the resistance to plastic indentation (H^3^/E_r_^2^) was calculated with the data obtained from the instrumented nanohardness [36]. The H^3^/E_r_^2^ ratio [37] is defined by the transition from elastic to plastic contact, proportional to the coatings type resistance to plastic deformation, and correlates well with wear resistance. Thus, the higher the H^3^/E_r_^2^ ratio, the greater the capacity of the coating to dissipate energy through plastic deformation during loading. As nitrogen flow rates increase, the H^3^/E_r_^2^ ratio increases, as seen in Figure 10.

**Figure 11:**
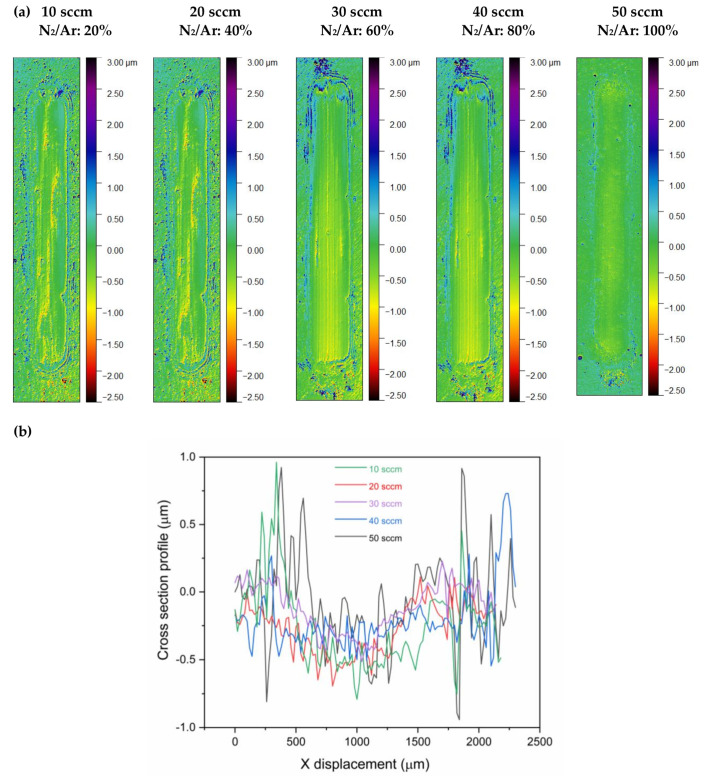
(**a**) Wear tracks are produced by linear reciprocating wear tests on the surface of (Cr, Y)N_x_ coatings as a function of the N_2_ flow rate. (**b**) Cross-section profiles.

The highest wear rate (1.92 × 10^−6^ mm3 N^−1^m^−1^) and wear volume (0.0096 ± 0.0015 mm³) were observed in (Cr, Y)N_x_ coatings formed under nitrogen flux below 20 sccm due to the lower hardness (19.0 ± 1.7 GPa) and larger granulometry of these coatings, as shown in Figure 10. Studies in Reference [38] reported a reduction in wear rate when reducing the grain size. Moreover, increasing nitrogen flow rates above 30 sccm decrease the wear rate from 0.513 × 10^−6^ to 0.130 × 10^−6^ mm^3^ N^−1^m^−1^ and the wear rate from 2.6 × 10^−3^ ± 0.9 × 10^−3^ to 0.6 × 10^−3^ ± 0.9 × 10^−3^ mm³, due to improvements in coating hardness (Cr,Y)N_x_ [39].

(Cr, Y)N_x_ coatings with higher N_2_ concentrations (30, 40, and 50 sccm) showed better performance in wear resistance (Figure 11), as well as the most increased coating toughness given by the H^3^/E^2^ ratios (Figure 10) [40]. Hence, we can observe the positive effects of the N_2_ flux on the phase composition, crystalline structure, and tribological and mechanical properties of multilayer-like (Cr, Y)N_x_ films produced by HiPIMS. The best mechanical and tribological properties were measured to thin films manufactured at the N_2_ flow equal to 50 sccm. According to Reference [41], increasing hardness was also verified for rising nitrogen contents in pure CrN films.

Figure 11 displays the wear tracks of (Cr, Y)N_x_ coatings generated by the dry linear reciprocating wear test, as measured by non-contact optical profilometry. All (Cr, Y)N_x_ coatings grown with different nitrogen flow rates showed material accumulation at the edges. The most significant wear track depth is for 10 sccm flow. As the flow of nitrogen increases, the maximum depths tend to decrease. The lowest maximum depth is for (Cr, Y)N_x_ films manufactured with a flow rate of 50 sccm [41]. Moreover, the grooves in the lower region of the indicative abrasive wear ranges are also notable.

**Figure 12:**
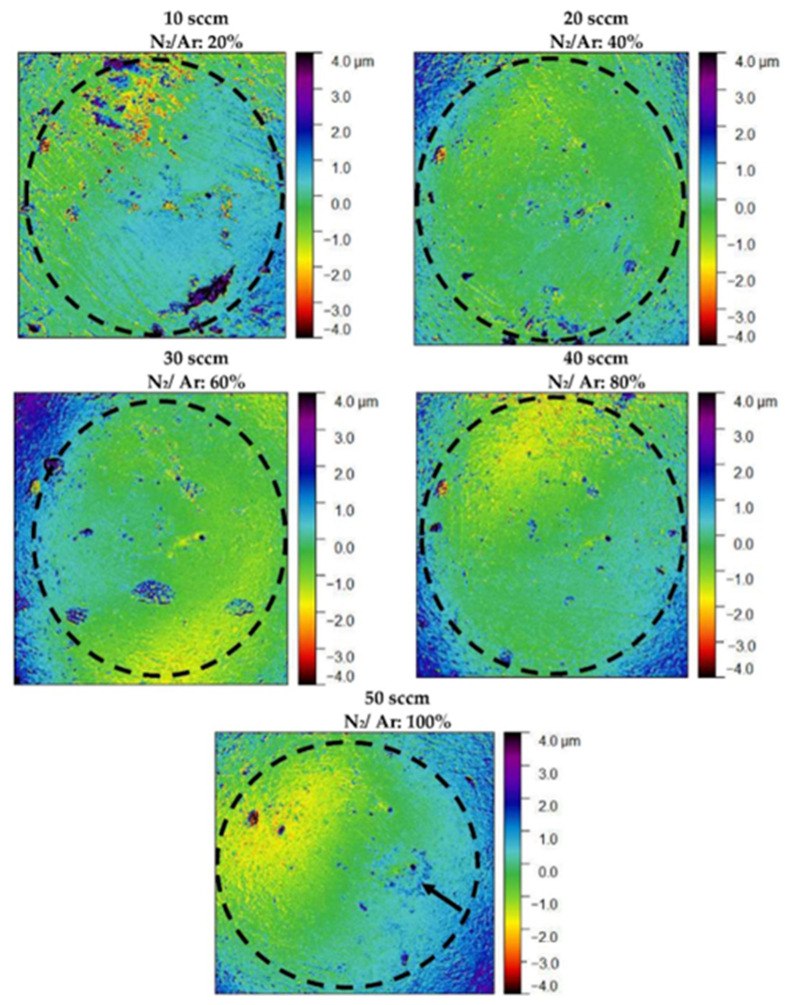
The surface of the Al_2_O_3_ ball counter body turned off against the (Cr, Y)N_x_ coating on which the theoretical bead was subtracted (dashed region indicating Al_2_O_3_ counter body). Black arrows indicate the adhered material.

Figure 12 displays the worn surface of the Al_2_O_3_ balls, presenting traces of adhered debris coming from the counter body. For Al_2_O_3_ balls in Figure 12, it is possible to observe material adhering to the surface, and for the 10 sccm flow rate, it is possible to follow wear marks, as expected from the abrasive wear mechanism. Note that the surface of the sphere with nitrogen fluxes of 10 and 20 sccm has the most material adhered [41,42].

The friction coefficient was measured during the dry reciprocating wear tests. Table 2 shows the maximum, and minimum peak friction coefficients (μ), as well as their root, mean square (RMS) values (μ_rms_), which are a statistical measure of the magnitude of friction along the tests. The multilayer-like (Cr, Y)N_x_ films manufactured with an N_2_ flux of 50 sccm exhibits a minimum μ_rms_ corresponding to the lowest wear rate [42]. This is possibly related to the reduced wear rate observed, which can be correlated with the obtained nanohardness values.

**Figure 13:**
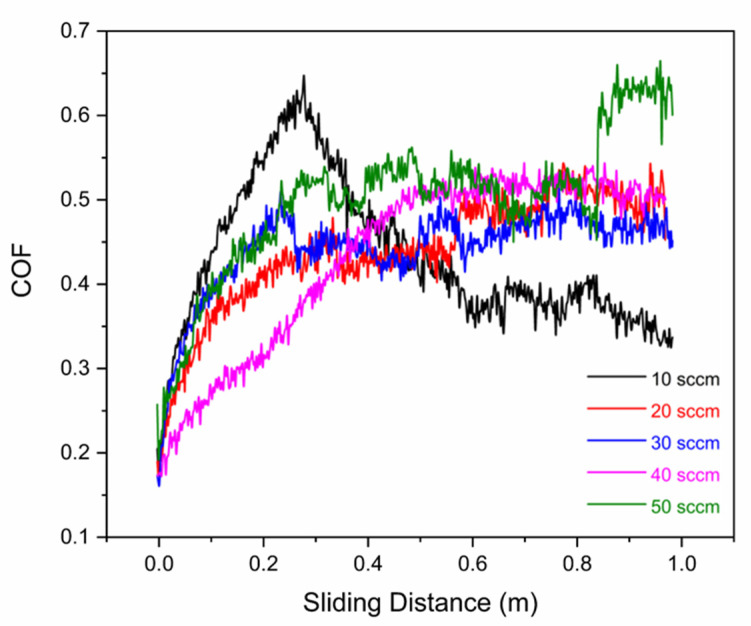
Coefficient of friction between the Al_2_O_3_ ball and the HiPIMS coatings as a function of N_2_ flow rate measured continuously along with the linear reciprocating wear tests.

When analyzing the friction coefficient of the (Cr, Y)N_x_ films, it is notable that the nitrogen flow of 30 sccm showed a rapid and sharp increase in the initial cycles, as shown in Figure 13. Coatings produced with a nitrogen flow of 10, 20, 40, and 50 sccm initially show a similar trajectory, with the coefficient of friction rising slower. However, for 10 sccm, there is a significant decrease in the friction coefficient. For flows of 20 and 40 sccm, the friction reaches a steady-state, while the coefficient of friction for the 50 sccm condition reaches a maximum steady-state value after a second gradual increase at the end of the cycle. 

## 4. Conclusions

Regarding the application of different nitrogen flow rates (10, 20, 30, 40, and 50 sccm) and the addition of 2 at.% yttrium on the microstructure, the following conclusions can be drawn:

1. The thickness and deposition rate increase as the nitrogen flow rate increases, consequent to the rise in HiPIMS peak voltages applied to the Cr-Y target.
2. The addition of 2 at.% Y increases the hardness of the fcc-CrN phase by almost 30% due to considerable refinement of the film architecture.
3. Phase formation at low nitrogen flux 10 to 30 sccm has a microstructure formed by fcc-CrN and hexagonal Cr_2_N; at higher nitrogen fluxes, there is only fcc-CrN formation.
4. The best mechanical and tribological performance was observed for the coating fabricated at a nitrogen flow rate of 50 sccm, where maximum hardness and wear resistance were obtained.

## Figures and Tables

**Table 1:** XRD data of the (Cr, Y)N_x_ films, such as plane and domain size.

Nitrogen Flow Rate	Phase	hkl	Domain Size (nm)
10 sccm	CrN	(111)	112 ± 1
		(200)	60 ± 1
		(331)	73 ± 1
		(440)	113 ± 1
	Cr_2_N	(111)	58± 1
		(300)	72 ± 1
20 sccm	CrN	(111)	62 ± 1
		(200)	57 ± 1
		(220)	53 ± 1
		(331)	105 ± 1
		(440)	312 ± 2
	Cr_2_N	(111)	57 ± 1
		(300)	131 ± 1
30 sccm	CrN	(200)	47 ± 1
		(331)	155 ± 1
		(440)	451 ± 2
	Cr_2_N	(111)	50 ± 1
40 sccm	CrN	(200)	71 ± 2
		(220)	57 ± 1
		(331)	231 ± 1
		(440)	431 ± 2
50 sccm	CrN	(111)	127 ± 1
		(200)	109 ± 2
		(220)	137 ± 1
		(331)	104 ± 1
		(440)	525 ± 2

**Table 2:** Coefficient of friction for multilayer-like (Cr, Y)N_x_ coatings sliding against Al_2_O_3_ balls, without lubricant, as a function of N_2_ flow rate during HiPIMS.

Nitrogen Flow Rate (sccm)	μ_rms_	μ_máx_
10	0.491	0.642
20	0.433	0.536
30	0430	0.510
40	0.425	0.537
50	0.420	0.659

## Data Availability

The original contributions presented in the study are included in the article; further inquiries can be directed to the corresponding author.
